# Effects of spinal manipulation combined with cervical kinetic chain training on neck muscle function in cervical vertigo: a preliminary prospective controlled study

**DOI:** 10.3389/fsurg.2026.1855125

**Published:** 2026-06-29

**Authors:** Bingyan Wang, Shuangyuan Xu, Xinyue Zheng, Shujia Liu, Shizheng Chen, Jiesheng Liu, Jiaxin Fu, Hehu Tang, Fangyong Wang, Junwei Zhang, Zhen Lyu

**Affiliations:** 1 School of Rehabilitation, Capital Medical University, Beijing, China; 2 Department of Spine and Spinal Cord Surgery, Beijing Bo'ai Hospital, China Rehabilitation Research Center, Beijing, China

**Keywords:** cervical muscles, cervical vertigo, cross-sectional area, dizziness, muscle hardness, randomized controlled trial, spinal manipulation

## Abstract

**Objective:**

Neck muscle dysfunction is associated with cervical vertigo. This study evaluated the clinical effect of “Spinal Manipulation Combined with Cervical Kinetic Chain Training” on neck muscle function in cervical vertigo.

**Methods:**

Data were collected from August 2023 to October 2024. 50 cervical vertigo patients were divided into a test group (*n* = 25) receiving spinal manipulation combined with cervical kinetic chain training, and a control group (*n* = 25) receiving conventional treatment. Assessments at baseline included vertigo severity (Visual Analog Scale, VAS; Dizziness Handicap Inventory, DHI), cervical muscle magnetic resonance imaging (MRI), and Shear Wave Elastography (SWE). Follow-ups at 4 weeks repeated VAS, DHI, and SWE. At 12 weeks, vertigo severity and muscle strength were assessed, with the test group undergoing repeat MRI.

**Results:**

DHI and VAS scores decreased significantly in both groups at 4 and 12 weeks compared to baseline. At 12 weeks, scores in the test group were significantly lower than at 4 weeks (*P* = 0.003), whereas the control group showed no difference (P_DHI_ = 0.677; P_VAS_ = 0.885). The test group's DHI was significantly lower than the control group's at 12 weeks (*P* = 0.001), and its VAS was lower at both 4 and 12 weeks. SWE showed the elastic modulus of bilateral splenius capitis muscles increased in the control group at 4 weeks (*P* left = 0.032; *P* right = 0.033), but remained unchanged in the test group (*P* > 0.05). At 4 weeks, the test group had a significantly lower elastic modulus in the right splenius capitis and obliquus capitis inferior than the control group (Right musculus splenius capitis, *P* = 0.031; Right obliquus capitis inferior, *P* = 0.031). At 12 weeks, the test group demonstrated a significant increase in the cross-sectional area (CSA) and oblique diameter compared to pre-treatment, the test group showed significant improvements in all the aforementioned metrics compared to baselines. The test group’s muscle strength of all directions were significantly better to those of control group.

**Conclusions:**

Spinal Manipulation Combined with Cervical Training enhances neck muscle strength, reduces suboccipital muscle tension compared to traditional treatment, and is an effective treatment for cervical vertigo.

**Clinical Trial Registration:**

https://www.chictr.org.cn/, Identiﬁer ChiCTR2300075144.

## 1 Introduction

Cervical vertigo is caused by changes in the position of the neck or diseases originating from the neck (1). It primarily presents with vertigo as the chief complaint, often with minimal physical signs that typically do not meet the indications for surgical intervention. Consequently, clinical management predominantly relies on conservative treatments, such as medication, acupuncture, manual therapy, and functional exercises (2–4). Although lacking clear surgical indications, cervical vertigo remains a prevalent orthopedic condition concerning spinal biomechanics and functional impairment. Therefore, providing an effective non-operative strategy is highly relevant to spinal surgeons. Recent studies have found that neck muscle dysfunction in patients with cervical vertigo is closely related to their vertigo symptoms (5). Compared with healthy individuals, patients show decreased stiffness in the anterior neck muscles, increased stiffness in the posterior neck muscles, and significantly reduced neck muscle strength. Furthermore, the severity of vertigo is correlated with the stiffness of the right obliquus capitis inferior and posterior extension strength. Therefore, improving neck muscle dysfunction through rehabilitation therapy may be a key component and effective treatment approach for alleviating cervical vertigo.

Spinal manipulation is a method that uses imaging guidance to locate misaligned vertebrae precisely and applies a controlled force at specific angles for correction (6); this method is also known as chiropractic manipulation. Following correction, the spinal vertebrae return to their anatomical position, restoring spinal curvature. This restoration allows muscle length, trajectory, and motor function to normalize, thereby enhancing spinal balance and stability (7, 8). Studies have shown that multiple spinal manipulation can significantly alleviate vertigo symptoms in patients. Andoni et al. utilized high-velocity low-amplitude chiropractic manipulation combined with massage, which significantly reduced the VAS and DHI scores postintervention (9). Micarelli et al. conducted a placebo-controlled trial in which ineffective laser therapy was used as a control, in which the Sustained Natural Apophyseal Glides (SNAGs) manipulation was administered to patients with cervical vertigo (10). Compared with the control group, the treatment group presented significant improvements in DHI scores, Neck Disability Index (NDI) scores, Neck Pain Index (NPI) scores, anxiety and depression scores, and cervical range of motion. The anatomical alignment of vertebrae is crucial for maintaining normal spinal biomechanics and coordinated muscle function. Prolonged vertebral misalignment can lead to the development of erroneous “muscle memory” in the surrounding muscles (11). Therefore, systematic functional training is required after manual adjustment to consolidate the therapeutic effects. Otherwise, without regular functional training to maintain the corrected position after manual adjustment, the muscles are prone to reverting habitually to their previous abnormal state.

The kinetic chain is a critical component for maintaining motor coordination and executing complex movements. Typical kinetic chain training exercises include deadlifts, planks, pull-ups, and suspension training (8, 12). Owing to its capacity to activate deep stabilizing muscles and enhance spinal stability, suspension training has been applied in the rehabilitation of patients with cervical spondylosis, and it has been shown to improve cervical range of motion and alleviate neck pain (13). Based on the aforementioned mechanisms, spinal manipulation aims to structurally correct misalignments, while kinetic chain training focuses on re-establishing long-term neuromuscular control and functional stability. The combination of the two therapies is likely to produce a synergistic effect. Building upon the foundation of Spinal Manipulation, this study incorporated a “cervical-scapular-upper extremity” kinetic chain training regimen. We aimed to investigate its therapeutic effects on symptoms, neck muscle strength, muscular imaging parameters, and muscle elasticity in patients with cervical vertigo, thereby providing a reference for understanding its pathogenesis and informing clinical management.

## 2 Materials and methods

### 2.1 Study design and participant enrollment

**Figure 1:**
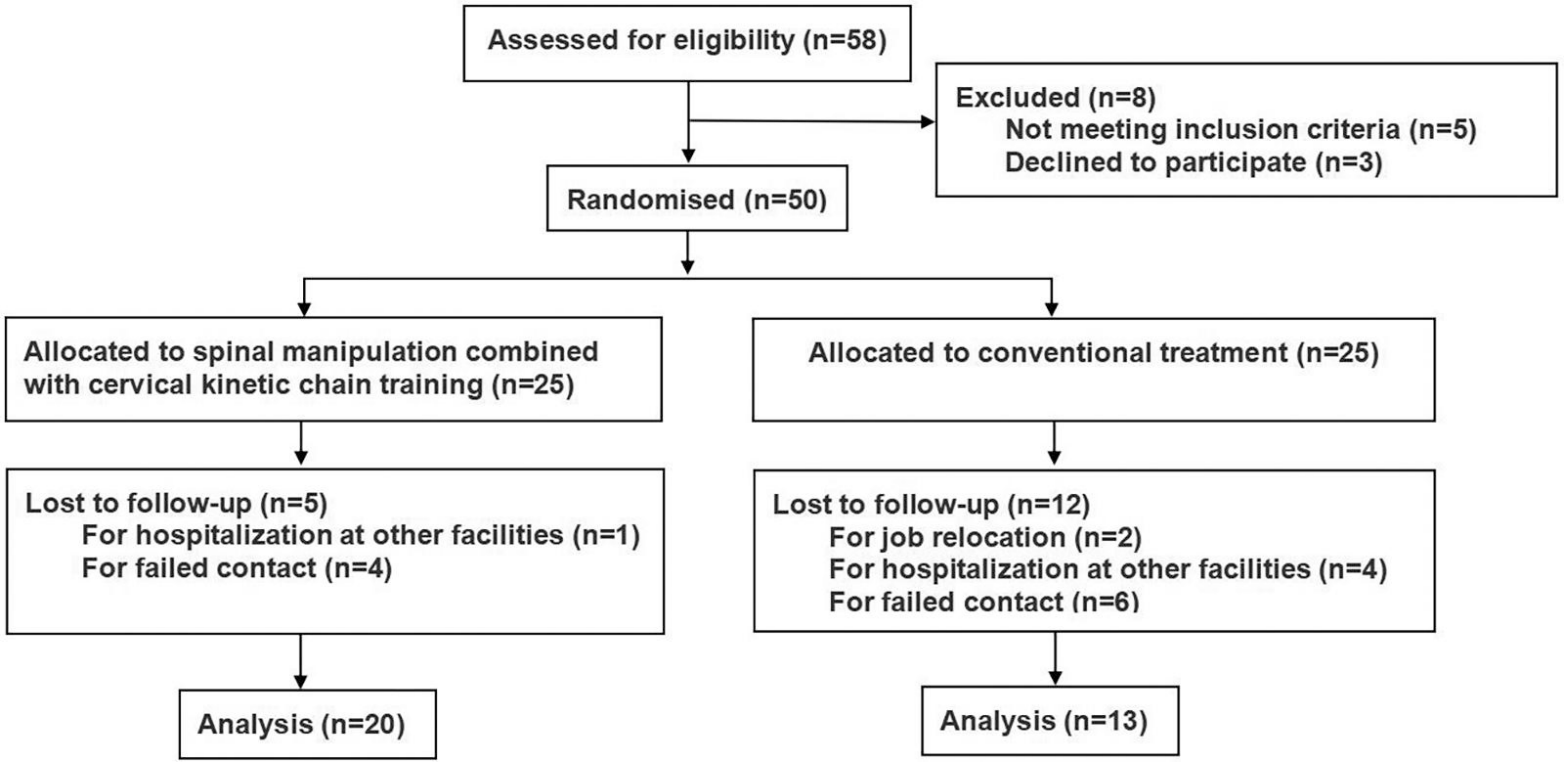
CONSORT flow diagram of the study.

This prospective, parallel-design, superiority randomized controlled trial enrolled patients with cervical vertigo aged 25–65 years, regardless of sex, who presented to the Department of Spine and Spinal Cord Surgery, Beijing Bo'ai Hospital, China Rehabilitation Research Center, between August 2023 and October 2024. A total of 50 participants were included and randomly assigned to either a test group (*n* = 25) or a control group (*n* = 25) (Figure 1). The main purpose of this study is to preliminarily verify the overall efficacy of this combined regimen compared to conventional therapy. Moreover, due to practical challenges in clinical implementation, it is difficult to achieve an evaluation of the therapeutic effects through multiple patient groups. Therefore, separate groups for spinal manipulation and kinetic chain training were not established.

The study was approved by the Ethics Committee of the China Rehabilitation Research Center (No. 2023-082-01). The trial was registered in the Chinese Clinical Trial Register (ChiCTR). The registration number is ChiCTR2300075144, date of registration is 26/08/2023. The study adhered to the CONSORT guidelines. All participants provided written informed consent. All procedures was conducted in accordance with the Declaration of Helsinki throughout the study period.

The inclusion criteria were as follows: (1) dizziness or vertigo accompanied by neck pain within 72 h; (2) dizziness or vertigo frequently provoked by neck movement; (3) a positive neck torsion test; (4) abnormal cervical imaging findings, such as cervical kyphosis, vertebral instability, or disc herniation; and (5) a history of neck trauma.

The exclusion criteria were as follows: (1) other types of vertigo, including benign paroxysmal positional vertigo, Ménière's disease, labyrinthine disorders, vestibular neuritis, ototoxic medication-induced vertigo, and motion sickness; central vestibular disorders (encompassing those resulting from cerebrovascular accidents or space-occupying lesions); ocular vertigo; vestibular symptoms secondary to systemic conditions (including circulatory dysfunction, hematological abnormalities, and metabolic intoxication); and dizziness secondary to neuropsychological disorders. (2) Poor patient compliance, specifically those unable to cooperate with completing the examinations and treatment follow-up. (3) Patients who were concurrently participating in treatment programs at other institutions.

Based on the primary outcome, the sample size was calculated using G*Power software. The calculation was based on the effect size derived from preliminary data on vertigo changes (a between-group difference of 12 in DHI scores). *A priori* power analysis indicated that at least 11 participants per group were required to detect a statistically significant difference (*α* = 0.05, 1-*β* = 0.80). To account for potential dropouts (estimated at 30% based on preliminary data), the final sample size was increased to at least 16 participants per group.

The implementation process of simple randomization is as follows: The random allocation sequence was generated by an independent statistician (who was not involved in subsequent subject recruitment, intervention implementation, or efficacy evaluation) using SPSS software version 26.0. The allocation scheme was placed into opaque, sealed envelopes, which were opened sequentially according to the order of subject enrollment. The researchers responsible for enrolling and assigning subjects were unaware of the random sequence. Subjects, efficacy assessors, and data analysts remained blinded to group allocation information. The entire randomization and blinding process adhered to clinical trial standards to minimize selection bias and information bias.

### 2.2 Outcome measurements

Vertigo Severity Assessment (14): The visual analog scale (VAS) score is used to quantify symptom severity and is rated from 0 to 10, with higher scores indicating more severe vertigo. The Dizziness Handicap Inventory (DHI) score was employed to evaluate the impact of vertigo on patients’ lives across physical, emotional, and functional domains, with a total score ranging from 0 to 100, where higher scores signify greater functional impairment.

**Figure 2:**
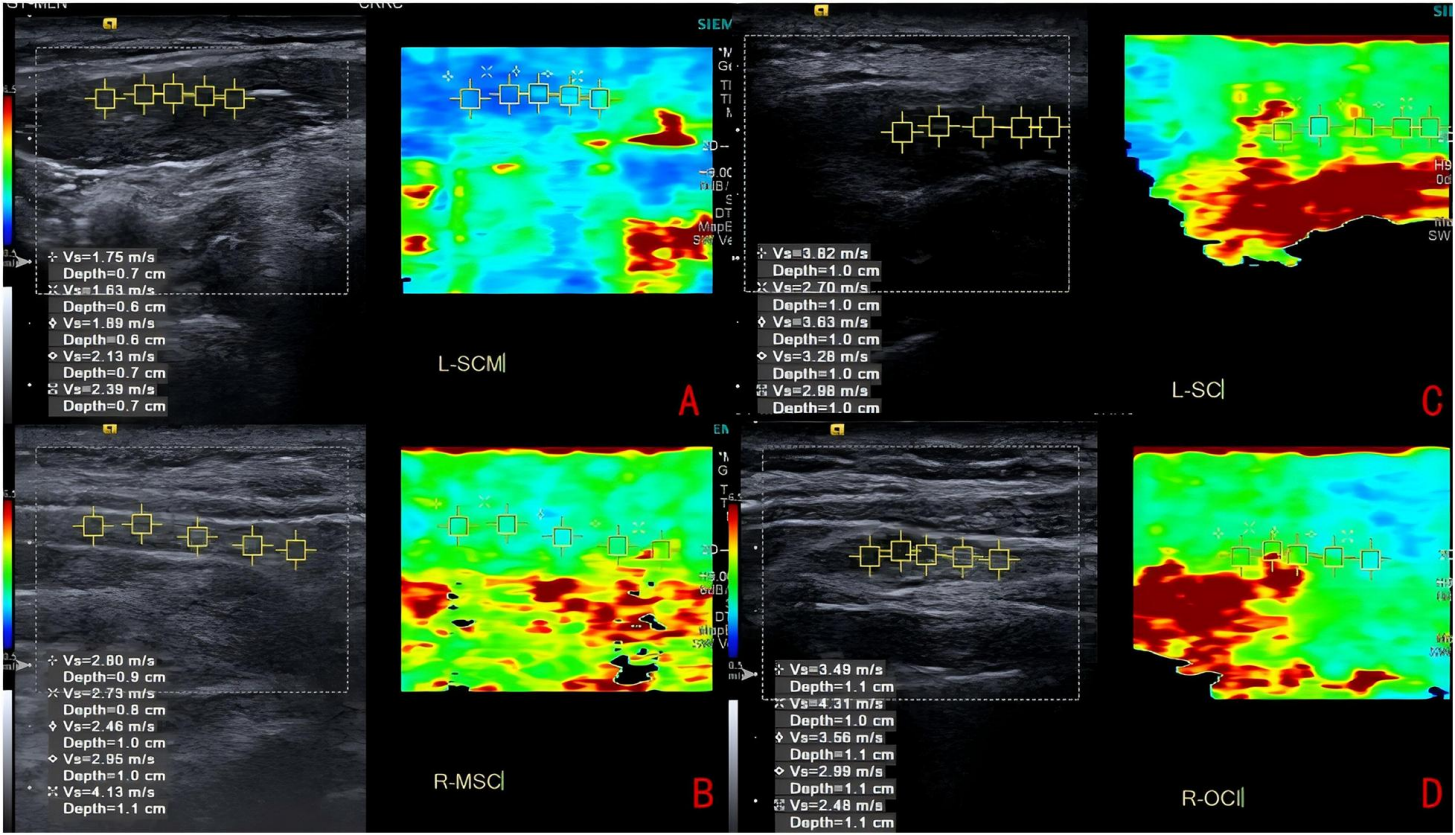
Patient's SWE examination. The figure is ultrasound elastography images of the neck muscles in a patient with cervical vertigo. Labels **(A–D)** denote the sternocleidomastoid, musculus splenius capitis, semispinalis capitis, and obliquus capitis inferior muscles, respectively. The grayscale images on the left demonstrate anatomical localization, while the corresponding images on the right display tissue elasticity characteristics. In the elastograms, red represents areas of high elastic modulus, and blue indicates areas of low elastic modulus. The continuous color transition from red to blue reflects a corresponding decrease in muscle fiber stiffness.

Quantitative Assessment of Neck Muscle Stiffness: (1) Equipment and parameters: A Siemens ACUSON OXANA2 ultrasound diagnostic system equipped with an ML6-15 linear array transducer (frequency bandwidth of 6–15 MHz) was used. (2) Measurement Procedure: The shear wave velocity (SWV, m/s) of the sternocleidomastoid, musculus splenius capitis, semispinalis capitis, and obliquus capitis inferior muscles was measured while the participants were in a seated position. This parameter is positively correlated with the elastic modulus (15). (3) Localization Method: The SWV of the sternocleidomastoid muscle was measured at the level of the C5 vertebral body/thyroid cartilage. Using the C7 spinous process as a bony landmark, the transducer was moved cephalad along the posterior midline to the C2 level and then moved horizontally laterally to identify and measure the SWV of the obliquus capitis inferior muscle (superior to the C2 lamina), semispinalis capitis muscle (superficial to the spinous process side), and musculus splenius capitis muscle (superficial to the transverse process side) (16, 17) (Figure 2). (4) Data collection: Five standard sites were selected for measurement of each muscle at rest, and the mean value was calculated after excluding data with motion artifacts. Blinded assessments (operators were unaware of group allocation) were performed by two physicians, each with over 10 years of experience in musculoskeletal ultrasound.

**Figure 3:**
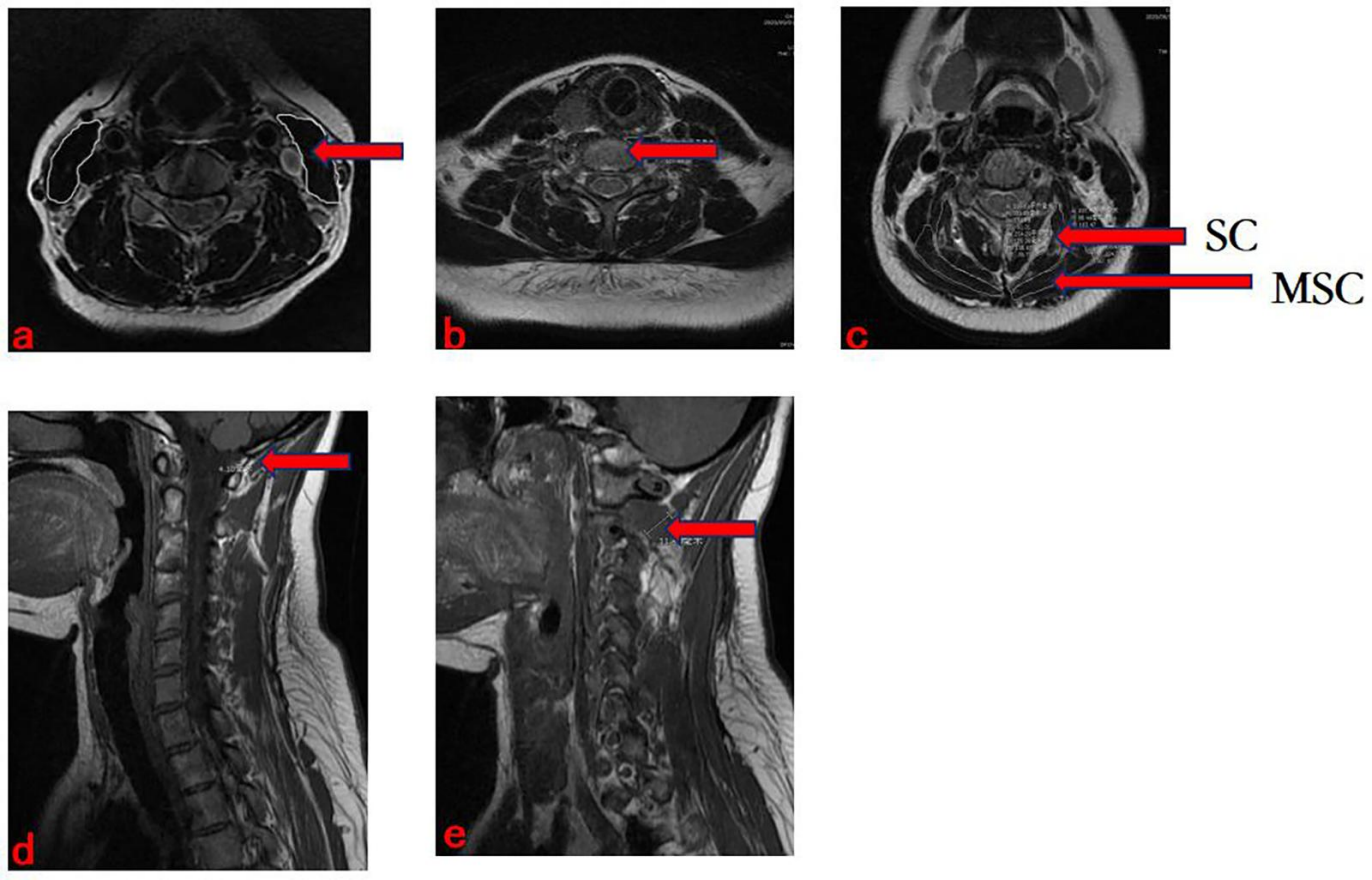
Muscle localization via neck MRI in a patient. **(a)** SCM(mid-level of C5 vertebral body); **(b)** LC(mid-level of C6 vertebral body); **(c)** MSC and SC(mid-level of C4 vertebral body); **(d)** RCPmi(lateral to the intervertebral joint, sagittal plane); **(e)** OCI(paraspinal, sagittal plane).

Quantitative assessment of neck muscle cross-sectional area and oblique diameter: (1) Equipment: A Philips Ingenia 3.0 T superconducting magnetic resonance imaging system and the Synex PACS medical image processing system were used. (2) Muscle Selection and Localization: The maximum cross-sectional area was measured for the longus colli (at the level of the C6 vertebral body), sternocleidomastoid (at the level of the C5 vertebral body), musculus splenius capitis, and semispinalis capitis muscles (at the level of the C4 vertebral body) (18, 19). The maximum oblique diameter was measured for the rectus capitis posterior minor muscle (in the sagittal plane lateral to the zygapophyseal joint) and the obliquus capitis inferior muscle (in the parasagittal plane adjacent to the spinous process) (20) (Figure 3). (3) Data acquisition: Manual delineation of the relevant muscle regions was independently performed by two radiologists. The system automatically calculates the area of the selected region. Each metric was measured twice, and the arithmetic mean was calculated.

**Figure 4:**
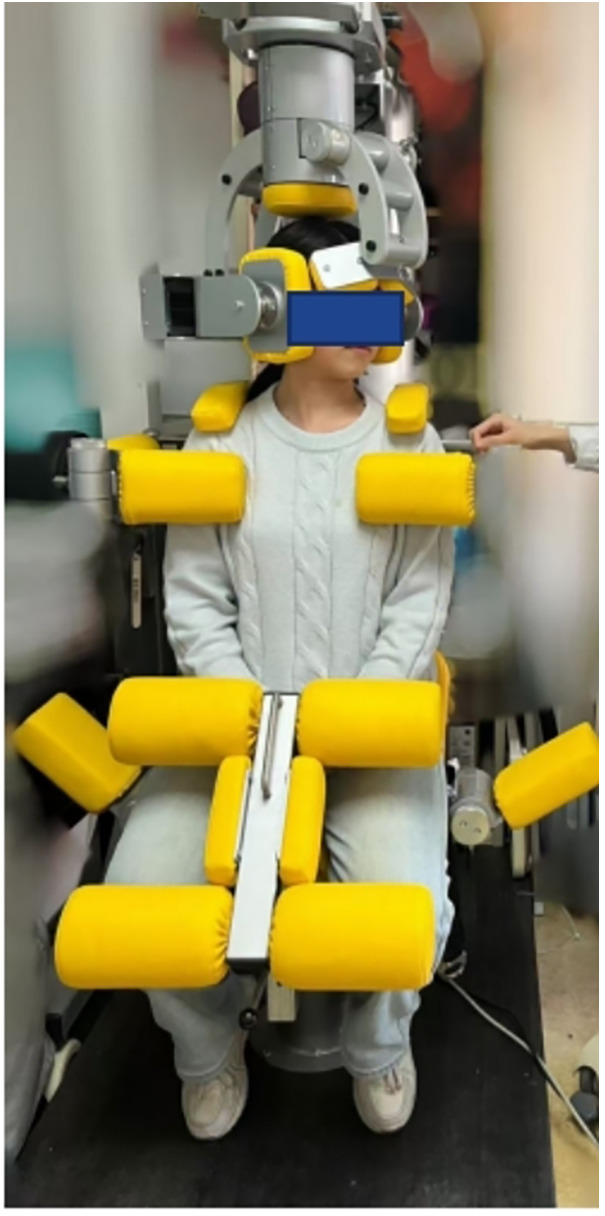
Assessment of mechanical parameters of neck muscles.

Cervical Spine Biomechanical Assessment: (1) Equipment: The Minotaur system (a three-dimensional measurement and training system for the neck and back), manufactured by BfMC Biofeedback Motor Control GmbH, Germany. (2) Test parameters: Cervical range of motion (recording the maximum left/right rotation angles) and maximum isometric contraction torque in various directions (flexion, extension, lateral flexion, rotation). (3) Testing Procedure: The subjects were seated and instructed to perform maximal effort resistance movements in each specified direction. Each direction was tested three times, and the mean value was calculated. (4) Data processing: Inertial forces were automatically eliminated by the system's built-in model, which subsequently generated standardized outputs (Figure 4).

Adverse Events: Following the intervention, inquiries and physical examinations were conducted to assess whether the intervention caused any discomfort, such as increased dizziness or pain, and the results were documented.

Assessment time: At baseline (pretreatment), both groups underwent evaluations for vertigo severity, cervical motor function, SWE, and MRI. At the 4-week treatment, vertigo severity and SWE were reassessed in both groups. At 12 weeks, vertigo severity and muscle strength were evaluated in both groups, with the test group undergoing an additional MRI assessment. Patients who failed to complete the 12-week treatment and follow-up were excluded from the final analysis, which included only those who completed the full study protocol.

### 2.3 Interventions

Patients in the control group received conventional treatment for 12 weeks, including medication, cervical collar immobilization, and self-administered neck exercises. Oral medications included Betahistine Tablets (6 mg, tid) and the proprietary Chinese medicine Xuan Yun Ning Tablets (1.14 g, tid). Patients were instructed to wear the cervical collar for 6 h per day while sitting or standing. Adherence was monitored through daily self-recorded wearing logs, which were reviewed and verified by the physician at each follow-up visit. The exercise regimen consisted of performing three sets per session, twice daily, with each movement sustained for 8–12 s and a 30-second rest between sets. Patients performed neck extensor strengthening exercises as follows: with hands clasped behind the head, neck extension was performed against the resistance provided by the hands. For lateral flexion training, one hand was placed against the temporal region above the ear, and lateral flexion was performed with the head pushing against the hand. The test group received Spinal Manipulation and kinetic chain training.

The Spinal Manipulation Protocol consists of three components: relaxation of the suboccipital muscles, cervical joint adjustment, and painful-point joint mobilization. Patients received 2 weeks of manual therapy in the initial phase, comprising a total of 4 sessions at a frequency of 2 sessions per week. (1) Suboccipital muscle relaxation: The patient lay in a supine position. The practitioner, seated at the head of the patient, gently applied axial traction by supporting the patient's mandible with one hand. On the other hand, with the thenar eminence supporting the occiput, the thumb and index finger were placed on the suboccipital muscles. The patient was instructed to slowly rotate their head while the practitioner performed relaxation techniques on these muscles. (2) Cervical Joint Adjustment: C2‒C6 Adjustment: The patient lay prone on a spinal manipulation table with Drop Mechanisms (Zhongjikang, Zhengzhou, China) with the head‒drop piece elevated. The patient's head was rotated 45° to one side. The practitioner fixed the lamina of the C2 to C6 vertebrae sequentially using the thenar eminence of one hand while applying a controlled, quick thrust with the other hand anterior to the patient's auricle. The head-drop piece was released simultaneously to facilitate joint adjustment. This procedure was performed bilaterally. Atlantoaxial (C1‒C2) Joint Adjustment: The patient lay supine on the spinal manipulation table. The practitioner palpated the transverse process of the atlas (C1) and assessed the direction of misalignment on the basis of clinical findings and open-mouth x-ray views. After local muscle relaxation, the patient's head was rotated to one side. The thumb of the superior hand fixed the transverse process of the atlas, whereas the other hand stabilized the contralateral mandible. A gentle, high-velocity, low-amplitude thrust was applied at the end range of rotation to adjust the atlas. (3) Painful-Point Joint Mobilization: The patient lay prone. The practitioner, positioned at the head of the bed, identified deviated spinous processes. The painful point on the convex side was selected. One thumb was placed on the root of the spinous process at the level of the pain point, whereas the other hand was fixed similarly on the contralateral side, caudal to the first contact point. Simultaneously, graded oscillatory pressure was applied toward the midline by both thumbs, and this process was repeated 2–3 times. The cervical spine was maintained in a neutral position throughout, and excessive force was avoided. All manipulations were performed by a licensed clinician with over 5 years of clinical experience, who had received specialized training and held certification in spinal manipulative therapy. The trial was conducted in strict adherence to the protocol, and participant compliance was moderate.

The kinetic chain training protocol is detailed in Table 1. For the initial training session, patients learned the training protocol under the guidance of a clinician, followed by subsequent home-based training. During home training, patients recorded their entire training sessions daily by taking videos and photos, which were sent to the clinician via mobile phone. This facilitated direct communication regarding training-related issues, allowing the clinician to provide timely corrections for any improper movements. Additionally, patients attended weekly in-person clinic visits to demonstrate their training progress, during which the clinician provided on-site guidance and corrected any improper movements.

**Table 1:** Kinetic chain training program.

Phase	Objective	Training content	Training intensity	Training frequency
Early (Within 1 week)	Increase joint range of motion; Prevent local adhesion	Supine position: Slow ROM maintenance training for flexion, extension, lateral flexion (L/R), and rotation (L/R) in cervical neutral position.	Hold each position 10–15s, rest 30s, 3 sets/session	2 times/day
Stability (1–3 weeks)	Enhance synergistic contraction of deep neck flexors and extensors; Improve dynamic cervical stability	Seated position: Isometric contraction for flexion, extension, lateral flexion (L/R), rotation (L/R) with light to moderate resistance (elastic band or manual resistance).	Hold each position 8–12s, rest 30s, 3 sets/session	2 times/day
Recovery (4–6 weeks)	Adapt to daily activities through functional training; Prevent recurrence	Standing Position: Perform cervical- scapular-upper extremity kinetic chain coordination training (including shoulder abduction with elbow flexion and forearm elevation; palms facing forward during upper limb abduction and arm lifting alongside the body, combined with active scapular retraction; resisted cervical extension and left/right cervical rotation assisted with elastic bands).	Hold each position 5s, rest 3s, Repeat 10 times per set, 3 sets/session	2 times/day

### 2.4 Statistical analysis

Statistical analysis was performed via SPSS version 26.0. Continuous data were first tested for normality using the Shapiro–Wilk test. Continuous data are presented as mean ± standard deviation (SD) if normally distributed or as Median (Q1, Q3) if non-normally distributed. Categorical data are presented as frequencies. Statistical analyses were performed based on the characteristics of the data. For continuous data conforming to a normal distribution, paired-sample t-tests were used for within-group comparisons, and independent-sample t-tests were used for between-group comparisons. For continuous data not conforming to a normal distribution, the Wilcoxon signed-rank test was used for within-group comparisons, and the Mann–Whitney U test was used for between-group comparisons. Fisher's exact test was used for between-group comparisons of categorical data. Overall differences between the two groups across multiple time points were analyzed using repeated-measures analysis of variance (ANOVA). Mauchly's test assessed the sphericity assumption, with Greenhouse–Geisser correction applied if violated. Due to a significant interaction effect between time and group(DHI: F = 6.905, *P* = 0.003; VAS: F = 10.896, *P* < 0.001), further simple effects analyses were conducted. Bonferroni correction was applied for multiple comparisons in the analysis of simple effects. A *P* value of less than 0.05 was considered statistically significant.

## 3 Results

### 3.1 Baseline characteristics

A total of fifty participants were enrolled in this study and randomly assigned, via a random number table method, to either the test group (*n* = 25) or the control group (*n* = 25). No adverse events related to the intervention were observed during the process. During the follow-up period, 17 participants were lost: 5 withdrew due to job relocation, 2 withdrew due to hospitalization at other facilities, and 10 were lost to follow-up because contact was unsuccessful. Consequently, 20 participants in the test group and 13 in the control group completed the study and were included in the final analysis. No significant differences were found in the baseline characteristics between the groups (Table 2). The test and control groups had similar sex distributions (5 M/15F vs. 4 M/9F; *P* = 0.716) and body mass index (BMI) (25.69 ± 5.03 vs. 25.02 ± 4.37 kg/m^2^; *P* = 0.700). The test group had higher mean DHI (40 ± 19 vs. 32 ± 16; *P* = 0.208) and pain VAS (7.05 ± 2.11 vs. 6.23 ± 1.30; *P* = 0.178) scores, but these differences were not statistically significant.

**Table 2:** Analysis of demographic characteristics in the test and control groups.

Variable	Test group	Control group	*P*-value
Age	42.45 ± 5.75	43.85 ± 8.18	0.568
Gender			0.716
Male	5	4	
Female	15	9	
BMI	25.69 ± 5.03	25.02 ± 4.37	0.700

Data are presented as mean ± standard deviation (SD) for continuous variables, and as frequency (n) for categorical variables.

### 3.2 Symptomatology assessment

**Figure 5:**
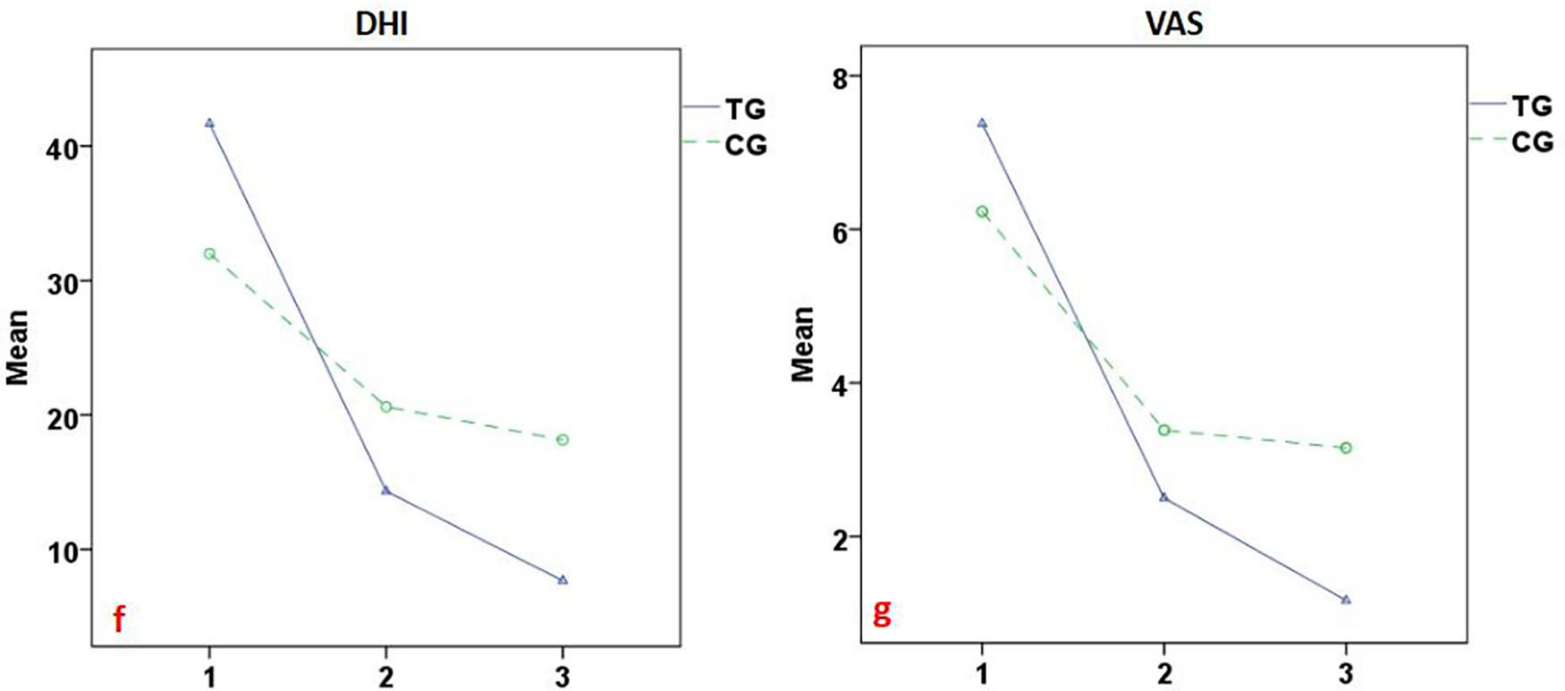
Trend of DHI and VAS scores at different time points in the test and control groups. Time point 1: pre-treatment; Time point 2: 4 weeks of treatment; Time point 3: 12 weeks of treatment.

Both the DHI and VAS scores significantly decreased from baseline at both the 4-week and 12-week time points in the test and control groups. In the test group, the scores at 12 weeks were significantly lower than those at 4 weeks (*P* < 0.05). In contrast, the control group showed no significant difference in scores between the 12-week and 4-week assessments (P_DHI = 0.677; P_VAS = 0.885) (Figure 5).

At the 4-week mark, the DHI scores of the test group were lower than those of the control group, but the difference was not statistically significant (*P* = 0.078). However, by 12 weeks, the DHI scores of the test group were significantly lower than those of the control group (*P* = 0.001). The VAS scores of the test group were significantly lower than those of the control group at both 4 weeks (*P* = 0.019) and 12 weeks (*P* < 0.001) (Table 3).

**Table 3:** Simple effects analysis of between-group differences in DHI and VAS scores.

					95% confidence interval for the difference	
Outcomes	Time point	Mean difference	Standard error	*P*-value	Lower bound	Upper bound
DHI	T0(G1-G0)	8.000	6.399	0.221	−5.050	21.050
	T1(G1-G0)	−9.515	5.217	0.078	−20.155	1.124
	T2(G1-G0)	−12.054	3.160	0.001*	−18.499	−5.608
VAS	T0(G1-G0)	0.819	0.656	0.221	−0.520	2.158
	T1(G1-G0)	−1.385	0.562	0.019*	−2.530	−0.239
	T2(G1-G0)	−2.204	0.464	<0.001*	−3.151	−1.257

T0: pre-treatment/baseline; T1: 4 weeks post-treatment; T2: 12 weeks post-treatment; G0: control group; G1: test group.

* a statistically significant difference.

### 3.3 Imaging assessment

**Figure 6:**
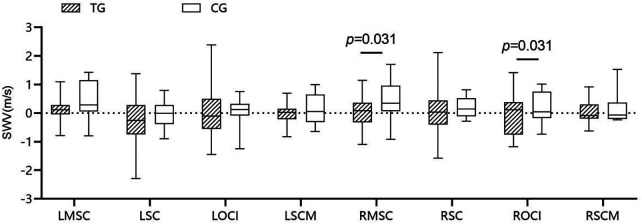
Between-group comparison of changes in muscle elastic modulus from pre- to post-treatment.

Ultrasound (elastic modulus): In the control group at 4 weeks post-treatment, the elastic modulus of the bilateral musculus splenius capitis muscles significantly increased compared with the pretreatment values (P_left = 0.033; P_right = 0.032). No significant differences were observed in the other measured muscles before and after treatment. In the test group, the elastic modulus of the bilateral musculus splenius capitis, semispinalis capitis, bilateral obliquus capitis inferior, and bilateral sternocleidomastoid muscles did not significantly change after 4 weeks of treatment compared with baseline (Table 4). In the between-group comparison, the elastic modulus of the right musculus splenius capitis and right obliquus capitis inferior muscles in the test group was significantly lower than that in the control group at the 4-week mark (Right musculus splenius capitis, *P* = 0.031; Right obliquus capitis inferior, *P* = 0.031) (Figure 6).

**Table 4:** Comparison of muscle elastic modulus in the test group pre-treatment versus post-treatment.

Muscle	Pre-treatment	Post-treatment	*P*-value
R MSC	2.87 (2.78, 3.22)	2.80 (2.63, 3.20)	0.717
R SC	3.69 ± 0.68	3.72 ± 0.59	0.884
R OCI	3.51 (3.05, 3.83)	3.31 (2.74, 3.55)	0.093
R SCM	1.93 (1.77, 2.20)	1.90 (1.73, 2.11)	0.411
L MSC	2.92 ± 0.46	3.03 ± 0.47	0.100
L SC	3.66 ± 0.72	3.43 ± 0.80	0.249
L OCI	3.34 ± 0.57	3.33 ± 0.78	0.962
L SCM	1.89 (1.78, 2.17)	1.93 (1.78, 1.99)	0.911

R: the right side; L: the left side; MSC, musculus splenius capitis; SC, semispinalis capitis; OCI, obliquus capitis inferior; SCM, sternocleidomastoid.

Data are presented as mean ± standard deviation (SD) if normally distributed, or as median (Q1, Q3) if non-normally distributed.

MRI: In the test group at 12 weeks post-treatment, significant increases in the cross-sectional area of the bilateral sternocleidomastoid, longus colli, musculus splenius capitis, and semispinalis capitis muscles, as well as in the oblique diameter of the bilateral rectus capitis posterior minor and obliquus capitis inferior muscles, were observed compared with the pretreatment measurements (Table 5).

**Table 5:** Comparison of muscle cross-sectional area and oblique diameter in the test group pre-treatment versus post-treatment.

Muscle	Pre-treatment	Post-treatment	*P*-value
R SCM	286.79 (246.20, 419.82)	328.38 (286.86, 437.29)	0.001*
L SCM	317.02 (268.89, 442.22)	343.17 (297.00, 455.70)	<0.001*
R LC	73.53 (59.86, 110.49)	89.74 (70.01.120.65)	<0.001*
L LC	88.39 ± 26.54	102.11 ± 28.52	<0.001*
R MSC	202.51 (178.56, 268.60)	218.36 (192.45, 283.78)	0.003*
L MSC	215.21 (169.03, 257.92)	233.20 (199.65, 289.06)	0.003*
R SC	219.98 (176.89, 301.34)	236.61 (192.79, 317.69)	<0.001*
L SC	248.96 (188.21, 303.63)	265.09 (204.05, 353.02)	0.001*
R RCPmi	3.75 ± 1.41	4.52 ± 1.89	0.006*
L RCPmi	3.96 ± 1.40	4.65 ± 1.47	0.003*
R OCI	14.33 (12.35, 16.33)	16.01 (13.78, 17.86)	<0.001*
L OCI	14.10 ± 2.94	15.43 ± 3.04	<0.001*

R: the right side; L: the left side; SCM, sternocleidomastoid; LC, longus colli; MSC, musculus splenius capitis; SC, semispinalis capitis; RCPmi, rectus capitis posterior minor; OCI, obliquus capitis inferior. Data are presented as mean ± standard deviation (SD) if normally distributed, or as median (Q1, Q3) if non-normally distributed.

* a statistically significant difference.

### 3.4 Kinematic assessment

At 12 weeks post-treatment, the control group showed no significant differences in cervical muscle strength (left/right rotation, flexion, extension, or left/right lateral flexion) or range of motion during left/right rotation (*P* > 0.05) (Table 6). Compared with pretreatment baseline, the test group demonstrated significant improvements at 12 weeks in both cervical muscle strength (left/right rotation, flexion, extension, and left/right lateral flexion) and range of motion in left/right rotation (Table 6). The between-group analysis revealed that, compared with the control group, the test group presented significantly greater improvements in cervical muscle strength (left/right rotation, flexion, extension, and left/right lateral flexion) and range of motion in left/right rotation at 12 weeks (Table 7).

**Table 6:** Comparison of cervical muscle strength and cervical range of motion within each group pre-treatment versus post-treatment.

Group	Indicator	Pre-treatment	Post-treatment	*P*-value
Control Group	left rotation (°)	58.40 (50.40, 63.20)	59.60 (51.30, 65.40)	0.600
	right rotation (°)	60.99 ± 12.91	62.39 ± 10.49	0.436
	Flexion (N)	5.00 (3.60, 6.60)	4.60 (3.40, 6.80)	0.421
	Extension (N)	8.70 (5.30, 13.90)	8.60 (5.90, 10.80)	0.381
	left lateral flexion (N)	5.20 (3.90, 7.80)	6.10 (4.10, 7.00)	0.366
	right lateral flexion (N)	5.20 (4.10, 7.40)	4.90 (4.00, 7.40)	0.727
	left rotation (N)	4.10 (2.00, 6.30)	4.30 (3.00, 6.50)	0.346
	right rotation (N)	4.60 (2.80, 6.90)	4.50 (2.70, 5.90)	0.700
Test Group	left rotation (°)	60.20 ± 5.40	71.9 ± 10.30	<0.001*
	right rotation (°)	63.90 (58.90, 69.90)	73.20 (66.60, 78.80)	0.001*
	flexion (N)	5.50 (3.70, 6.20)	7.80 (6.40, 9.10)	0.004*
	extension (N)	9.10 (6.50, 12.20)	12.40 (10.70, 14.20)	0.009*
	left lateral flexion (N)	5.90 (4.50, 7.70)	8.10 (6.40, 10.60)	0.004*
	right lateral flexion (N)	6.70 (4.70, 9.50)	9.40 (7.50, 11.60)	0.001*
	left rotation (N)	4.70 (4.10, 6.90)	7.30 (5.50, 8.60)	<0.001*
	right rotation (N)	5.40 (4.50, 7.70)	7.60 (6.30, 9.00)	0.001*

Data are presented as mean ± standard deviation (SD) if normally distributed, or as median (Q1, Q3) if non-normally distributed.

* a statistically significant difference.

**Table 7:** Comparison of the differences in cervical muscle strength and cervical range of motion between the two groups pre-treatment versus post-treatment.

Indicator	Test group	Control group	*P*-value
left rotation (°)	11.73 ± 10.02	0.72 ± 3.95	<0.001*
right rotation (°)	8.87 ± 7.75	1.40 ± 6.28	0.007*
Flexion (N)	2.45 (0.85, 3.73)	0.80 (−1.10, 1.10)	0.009*
Extension (N)	2.15 (−0.38, 5.10)	−0.10 (−0.85, 0.35)	0.019*
left lateral flexion (N)	2.05 ± 2.43	−0.36 ± 1.60	0.004*
right lateral flexion (N)	2.43 ± 2.53	0.28 ± 2.01	0.015*
left rotation (N)	1.80 (0.53, 2.75)	0.00 (−0.25, 1.00)	0.013*
right rotation (N)	1.65 (0.65, 2.75)	−0.20 (−0.40, 1.10)	0.007*

Data are presented as mean ± standard deviation (SD) if normally distributed, or as median (Q1, Q3) if non-normally distributed.

* a statistically significant difference.

## 4 Discussion

Cervical vertigo is currently widely believed to be associated with cervical pathologies. There are six primary pathogenic theories: proprioceptive afferent abnormalities (21), sympathetic irritation (22), vertebral artery insufficiency (23), cervical instability (24), humoral factors (25), and migraine-related factors (26). These mechanisms often coexist and interact rather than operating in isolation (27). Notably, most theories involve cervical muscle dysfunction (28). Chronic strain of muscles and fascia may play a critical role in the development of chronic nonspecific neck pain (29), and since cervical vertigo and neck pain frequently cooccur, their pathogenesis may share common mechanisms involving functional abnormalities of the cervical muscles and fascia.

This study revealed that both DHI and VAS scores significantly improved after treatment in both groups compared with pretreatment levels, with the test group demonstrating superior improvement over the control group, particularly in VAS scores. These findings indicate that the therapeutic approach employed in this study achieves favorable clinical efficacy in alleviating symptoms of cervical vertigo. Ning et al. applied Long's manipulation for the treatment of cervical vertigo and reported significant improvements in vertigo severity, frequency, and duration (30). The spinal manipulation therapy employed in the studies by Andoni et al. (9) and Micarelli et al. (10) also significantly alleviated cervical vertigo and improved cervical dysfunction.

In this study, the test group demonstrated significantly lower VAS scores than did the control group at both 4 and 12 weeks, whereas significant between-group differences in DHI scores emerged only at the 12-week assessment. This temporal discrepancy in DHI improvement may be attributed to its multidimensional assessment nature. Owing to individual physiological variations, recovery across different domains progresses asynchronously: physical function may improve rapidly, whereas emotional regulation and adaptive capacity may recover more slowly. This heterogeneous recovery pattern explains the delayed DHI response compared with the immediate symptom improvement captured by the VAS. Both the DHI and VAS scores significantly improved from 4 to 12 weeks in the test group, whereas no statistically significant changes were detected in the control group. The possible reason for this is that the control regimen (mechanical stabilization and pharmacotherapy) provides short-term symptom reduction but is associated with poor long-term adherence to medication and cervical collar use. Furthermore, prolonged collar immobilization may exacerbate disuse atrophy of neck muscles, leading to decreased joint mobility and diminished strength, potentially worsening muscular dysfunction. In contrast, the test group received combined manual therapy and kinetic chain training. This comprehensive approach corrected pathological cervical biomechanical alignment, restored physiological curvature, and implemented targeted strengthening exercises for neck musculature. Consequently, the long-term effect is better.

The cervical muscles are essential for maintaining spinal dynamic equilibrium and movement stability (31), whereas the cervical ligaments support only approximately 2 kg of static load, with dynamic loads managed through muscular cocontraction (32). Exercise therapy has gained recognition for its ability to manage cervical spondylosis symptoms, particularly resistance training, because of its significant therapeutic effects. However, relatively few studies on resistance training in the treatment of cervical vertigo exist. In a study of 50 patients with cervical vertigo, Fan et al. demonstrated that specialized resistance training improved vertigo severity and neck disability index scores. Imaging suggested that the underlying mechanism might involve increased deep muscle strength and restored physiological curvature (33). Our findings corroborate this finding, showing that kinetic chain resistance training significantly improved muscle strength in all movement directions alongside substantial vertigo reduction, indicating a clear relationship between strength gains and symptom improvement. The primary pathogenesis of cervical vertigo involves vertebral artery compression or spasm, leading to cerebral blood flow insufficiency resulting from misalignments of the atlantoaxial and facet joints (24, 34, 35). Anderst et al. reported that high-velocity, low-amplitude manipulation significantly increased the intervertebral range of motion across multiple cervical segments (36). Further research has indicated that while exercise therapy alone might reduce cervical mobility, the combination of manual therapy and exercise can effectively increase the cervical range of motion (37). Our integrated approach successfully restored joint alignment, increased mobility, and reduced vertigo symptoms. This therapeutic effect may be attributed to manual adjustments restoring cervical curvature and joint positioning while normalizing muscular tension, complemented by kinetic chain training that enhances the neuromuscular function of the cervical muscles. This combined approach synergistically improves cervical stability from both structural and dynamic perspectives, consequently achieving sustained symptomatic relief. This suggests that the therapeutic benefit arises from the synergy between structural correction via spinal manipulation and neuromuscular function enhancement via kinetic chain training, rather than the isolated effect of either component alone.

Compared with the control group, the test group presented a significantly lower elastic modulus in the right musculus splenius capitis and obliquus capitis inferior muscles posttreatment, whereas the control group presented an increased elastic modulus in the bilateral musculus splenius capitis muscles. Muscle elasticity depends primarily on fiber type composition and connective tissue. Fast-twitch fibers generate rapid, high-force contractions but fatigue quickly, whereas slow-twitch fibers produce sustained, endurance-oriented contractions with better fatigue resistance. Research indicates that individuals with higher slow-twitch fiber proportions demonstrate superior elastic energy utilization (38). Correspondingly, patients with cervical instability have reduced slow-twitch fiber percentages in deep posterior neck muscles (39). In our test group, the observed improvement in elasticity likely reflects kinetic chain training-induced slow-twitch fiber adaptation, whereas increases in stiffness in the control group suggest fast-twitch fiber dominance. Furthermore, the regulation of muscular tension is another critical factor influencing elasticity. Normal muscles maintain optimal tension, whereas overstretching causes dysregulation and stiffness. Our intervention restored cervical curvature and joint alignment, alleviating muscular overstretching, whereas concurrent kinetic chain training strengthened the weakened musculature. This dual approach normalized muscular tension and reduced the elastic modulus. Nevertheless, the precise underlying mechanisms warrant further investigation.

The test group presented significant increases in muscle cross-sectional area and oblique diameter posttreatment. This aligns with established correlations between the cross-sectional area of the cervical muscle and muscle strength (40, 41). The muscle cross-sectional area is influenced by the number, type, and size of muscle fibers, as well as the composition of other soft tissue components. As muscle fiber quantity remains constant in adults, the observed hypertrophy suggests two mechanisms: (1) exercise-induced fiber enlargement enhances strength and vertigo relief, and (2) manual therapy-exercise interventions modify soft tissue composition to increase the cross-sectional area and improve symptoms. These morphological changes are positively correlated with neck strength enhancement. This morphological adaptation likely results from resistance training-stimulated muscle fiber hypertrophy, consequently augmenting muscle power—a phenomenon that is consistent with clinical observations.

The suboccipital muscles, which are rich in muscle spindles, are vital for head‒neck control and proprioception (42). This study revealed a significantly increased oblique diameter in key suboccipital muscles posttreatment. This structural adaptation from kinetic chain training enhances rotational strength, providing both a physiological basis for strength improvement and objective evidence for superior cervical stability.

Due to the epidemiological characteristics of cervical vertigo, female patients constitute the vast majority of patients. Consequently, our cohort was predominantly female. The limited sample size precluded gender stratification; thus, these findings should be interpreted as preliminary results. Additionally, the narrow age range (mean ages 42–43, SD 5–8 years) rendered age-stratified analysis unnecessary. We fully acknowledge this limitation. In future research, we will expand the sample size and recruit more male participants to conduct a more in-depth analysis of how demographic characteristics influence treatment outcomes.

The study limitations include a small sample size, short symptom history in most patients, and a 3-month follow-up period that is insufficient for assessing long-term outcomes. Owing to budget constraints, the control group lacked 12-week MRI data. The dropout rate in the study was relatively high, as many patients showed a significant decline in adherence and were unable to persist with the treatment protocol once their vertigo symptoms improved, resulting in a further reduction in the effective sample size for analysis. Furthermore, as noted earlier, the absence of independent groups for spinal manipulation and kinetic chain training, coupled with the lack of subgroup analysis, are also limitations of this study. Future studies should expand the cohort size, establish independent groups for spinal manipulation and kinetic chain training, extend the treatment duration, and incorporate multiple follow-ups. Moreover, demographic subgroup analyses should be conducted to further validate the sustained efficacy of the combined therapy and identify the populations that derive the greatest benefit.

## 5 Conclusion

Cervical vertigo patients exhibit muscle dysfunction characterized by weakness, stiffness, and restricted mobility, particularly in the cervical extension and suboccipital muscles. While conventional treatments provide symptom relief, combined spinal manipulation and kinetic chain training demonstrate superior efficacy. Considering the limited sample size of this study, the preliminary findings suggest that the integrated approach can significantly alleviate vertigo, reduce muscle stiffness, increase the muscle cross-sectional area, and enhance neck muscle strength. Therefore, this combined approach may be recommended as a valuable and applicable therapeutic strategy for the clinical management of cervical vertigo.

## Data Availability

The original contributions presented in the study are included in the article/Supplementary Material, further inquiries can be directed to the corresponding author.
